# Review of Minimally Invasive Esophagectomy and Current Controversies

**DOI:** 10.1155/2012/683213

**Published:** 2012-08-02

**Authors:** T. Kim, S. N. Hochwald, G. A. Sarosi, A. M. Caban, G. Rossidis, K. Ben-David

**Affiliations:** Department of Surgery, College of Medicine, University of Florida, P.O. Box 100109, Gainesville, FL 32610-0109, USA

## Abstract

Esophagectomy is a complex operation with significant morbidity and mortality. Minimally invasive esophagectomy (MIE) was described in the 1990s in an effort to reduce operative morbidity. Since then many institutions have adopted and described their series with this technique. This paper reviews the literature on the variety of MIE techniques, clinical and quality of life outcomes with open versus MIE, and controversies surrounding MIE—such as prone positioning, stapling techniques, size of the gastric conduit, and robotic techniques.

## 1. Introduction

Esophagectomy for benign or malignant disease is a complex operation with significant morbidity and mortality. The five-year survival rate has been shown to be 19% to 46.5%, with a surveillance program and increased detection of early-stage esophageal cancer [1–3]. Despite these survival rates, Orringer and his colleagues set the benchmark for postoperative outcomes with their reported series of 1,085 patients who underwent open transhiatal esophagectomy from 1976 to 1998 with a hospital mortality and morbidity rate of 4% and 13%, respectively [4]. More recently, they published an updated series of 2,007 patients who had an associated hospital mortality rate of 1%, anastomotic leak rate of 9%, pneumonia rate of 2%, and intrathoracic hemorrhage, recurrent laryngeal nerve paralysis, chylothorax, and tracheal laceration rates of <1% each [5]. 

In an effort to reduce the morbidity of esophagectomy, minimally invasive methods began to be increasingly applied in the 1990s. In 1998, Luketich et al. reported their initial experience on 8 patients who underwent minimally invasive esophagectomy (MIE) using either laparoscopic and/or thoracoscopic techniques. They had no perioperative mortalities and one cervical anastomotic leak, thus demonstrating the potential safety and feasibility of minimally invasive esophagectomy [6]. Since then, many institutions have reported their experiences in the adoption and refinement of MIE for benign and malignant diseases of the esophagus. Hence, this paper summarizes the literature to date, including both larger single institution series on MIE and also retrospective and prospective comparisons of open versus MIE, and addresses the various lessons learned and controversies surrounding MIE.

## 2. Various Techniques of Esophagectomy

The two most commonly employed open techniques of esophagectomy are transhiatal esophagectomy (THE) and Ivor Lewis esophagectomy (ILE). First described in 1936, THE involves laparotomy with blunt dissection of the esophagus and cervical esophagogastric anastomosis [7]. ILE involves combined laparotomy with right thoracotomy and intrathoracic anastomosis [8]. Other approaches include resection via left thoracotomy or left thoracoabdominal approach or three-incision McKeown-type esophagectomy [1, 2, 4, 9, 10]. In a previous report by Pennathur et al. in 2010, the various open techniques were described along with a few comparative studies on open versus MIE [11, 12]. For the purpose of this paper, open techniques of esophagectomy include any of the above procedures via completely open approach (without any laparoscopic or thoracoscopic component): THE, ILE, esophagectomy via left thoracotomy or left thoracoabdominal approach; MIE techniques include both total laparoscopic/thoracoscopic THE or ILE and also hybrid procedures with at least one of the approaches being done via either laparoscopy or thoracoscopy. Summarized in a review by Herbella, the various techniques of MIE include any combination of laparoscopy instead of laparotomy, thoracoscopy instead of thoracotomy, and either cervical (THE) or intrathoracic (ILE) anastomosis [13].

As with many new novel procedures, the initial publications involving minimally invasive esophagectomy were mostly institutional series. Outcomes from these institutional series included anastomotic leak rates of 4% to 11.7%, pneumonia rates of 7.7% to 16.7%, major morbidity rates of 12.5% to 23%, and operative mortality rates of 0.9% to 6% (Table 1).

Luketich, one of the earlier pioneers of MIE, reported his extensive experience from 1996 to 2002 on 222 patients who underwent MIE for either high-grade dysplasia (*n* = 47) or invasive cancer (*n* = 175). MIE was successfully completed in 206 (92.8%) patients. Operative mortality was 1.4%. Morbidity included anastomotic leak rate of 11.7%, pneumonia incidence of 7.7%, and recurrent laryngeal nerve injury with vocal cord palsy rate of 3.6% [14]. Their preferred and most commonly employed approach is combined right thoracoscopic and laparoscopic THE. If significant gastric extension of the tumor is encountered, they prefer to resect more stomach and perform an intrathoracic anastomosis, that is minimally invasive ILE. The same group later described their early experience with minimally invasive ILE in 50 patients from 2002 to 2005 with an operative mortality and leak rate of 6% each [15].

Rajan et al. also published a large series of 463 patients in India who underwent minimally invasive esophagectomy from 1997 to 2009. Interestingly, 71 percent of patients had squamous cell carcinoma, and 29% had adenocarcinoma of the esophagus. Operative mortality was 0.9%, and overall morbidity was 16% [16]. Similarly, Nguyen and colleagues reported a series of 104 MIE procedures performed between 1998 and 2007. Most procedures were minimally invasive THE or ILE (98 of 104) utilizing a circular staple technique.

Major complication rate was 12.5%, and minor complication rate was 15.4%. Anastomotic leak rate was 9.6%, and operative mortality was 2.9% [17]. Consequently, we reported our results in 105 consecutive patients who underwent MIE utilizing a side-to-side 6 cm linear stapled technique from August 2007 to January 2011. Our mortality was 1% (1/105), and morbidity included 7% transient left recurrent laryngeal nerve injury, 9% pneumonia, 1% wound infection, and 4% anastomotic leak rate [18]. In a separate study, we studied the effect of neoadjuvant chemoradiation on outcomes after MIE and noted that there were no significant differences in operative blood loss, median operative time, total or individual complication rates, pneumonia, atrial fibrillation, recurrent laryngeal nerve injury, or anastomotic leaks between patients who received neoadjuvant chemoradiation and patients who did not [19]. Finally, due to the proximity of our Veterans Administration Hospital to the University Hospital, we were able to demonstrate the feasibility of an MIE program at the Veterans Hospital in our initial series of 18 consecutive MIE. There was one (5.6%) 30-day mortality, 1 (5.6%) anastomotic leak, and 3 (16.7%) postoperative pneumonias [27]. 

In 2007, Gemmill and McCulloch published one of the earlier systematic reviews of minimally invasive operations for esophageal and gastric cancer based on an electronic search of the literature from 1997 to 2007. From 188 abstracts reviewed, 23 articles were found on minimally invasive esophagectomies (*n* = 1398)—the operations spanning any combination of thoracoscopy or thoracotomy with laparoscopy, hand-assisted laparoscopy, or laparotomy (i.e., MIE or hybrid MIE). Twenty-one of the 23 were case studies, and the remaining 2 were case-matched studies; there were no randomized controlled studies of open versus MIE at the time of this systematic review. For MIE or hybrid MIE, 30-day mortality was 2.3%, combined major and minor morbidity was 46.2%, anastomotic leak rate was 7.7%, and respiratory tract infection rate was 13.2%. The authors stated that while there appears to be substantial literature suggesting the feasibility and safety of minimally invasive surgery for esophageal cancer, the quality of the studies was poor [20]. Flaws included (1) the predominance of case series—low levels of evidence, (2) lack of valid direct comparisons of open versus MIE, (3) heterogeneity of the studies with regard to the type of MIE or hybrid MIE and, thus, lack of generalizability, (4) selection bias—patients selected for minimally invasive surgery are unlikely to have been representative of the general population of esophageal cancer patients (i.e., earlier stage, smaller tumors, and/or less co-morbid), and (5) publication bias—surgeons with unsatisfactory results may have been less inclined to publish their data. Hence, the authors suggested a prospective nonrandomized cooperative study by surgeons interested in first establishing a consensus on both the appropriate question and the appropriate procedure to be tested against open esophagectomy. The study would allow for evaluation of learning curves, power calculations based on observed treatment effect, and development of quality measures; the study would thus serves as an important and less costly preliminary step before a randomized controlled trial.

Verhage et al. published their results of a systematic review consisting of 10 case-control studies comparing open to MIE. Blood loss for MIE (compared to open esophagectomy) was uniformly lower in all studies, whereas hospital and ICU length of stay, total complication rate, and pulmonary complications were significantly lower with MIE in most studies [21]. This paper was limited by the heterogeneity of the studies with regards to technique of MIE and both selection and publication bias. A meta-analysis by Nagpal et al., consisting of 12 studies comparing open esophagectomy (*n* = 612) and MIE or hybrid MIE (*n* = 672), concluded similar findings as noted by Verhage's group. There were no significant differences in 30-day mortality or anastomotic leak rates. Blood loss, ICU length of stay, overall hospital stay, and total morbidity were significantly lower in the MIE group [22].

A more recent systematic review by Dantoc et al. comparing open to MIE consisted of 17 case-control studies, and the review showed no significant differences in 30-day survival or 5-year survival rates. Median number of lymph nodes retrieved was significantly higher with MIE versus open esophagectomy (16 versus 10) attributed to better visualization with MIE [23]. Furthermore, in a meta-analysis by Sgourakis et al., 8 out of 71 screened trials comparing open versus MIE or hybrid MIE were included in the final study (*n* = 1,008). It was found that total complications were lower with MIE (odds ratio or OR 1.93, 95% confidence interval or CI 1.08–3.43 for open versus MIE). It should be noted, however, that this comparison was performed with only 3 studies. Anastomotic stricture rates were lower with open esophagectomy (OR 0.11, 95% CI 0.04–0.31), but this comparison was performed with only 2 studies [24]. Additionally, Biere et al. published their findings of a meta-analysis in which 1 controlled clinical trial and 9 case-control studies were included in the final study (*n* = 1,061). Trends were observed in favor of MIE for the following outcomes: major morbidity, pulmonary complications, anastomotic leakage, mortality, length of stay, operating time, and blood loss, but statistical significance was not reached [25]. The obvious limitation of these meta-analyses is that they consist of primarily nonrandomized and retrospective case-control studies. Ultimately, it was concluded in all three meta-analyses that prospective randomized controlled trials comparing open versus MIE were needed.

Lastly, a large United Kingdom population-based study by Mamidanna et al. analyzed the Hospital Episode Statistics data from April 2005 to March 2010 and included 7,502 esophagectomies, 1,155 (15.4%) of which were MIE—with marked increase in the proportion of MIE (24.7%) performed from 2009-2010. There was no difference between open and MIE groups, respectively, in 30-day mortality (4.3% versus 4.0%) and overall morbidity (38.0% versus 39.2%). Reintervention rate was higher with MIE compared to open (21% versus 17.6%, *P* = 0.006) [26]. Despite the seeming equipoise in outcomes between open and MIE in the above study, there were significant limitations of this population-based study based on an administrative database, as commented in editorials by Rice and Blackstone [28] and Pennathur and Luketich [29].

Based on these large studies on MIE, total complication rates range from 38% to 46.2% and operative mortality rates range from 1.3% to 4.3% (Table 1). The numerous studies comparing open versus MIE (case-control studies and meta-analyses or systematic reviews) suggest that oncologic outcome and survival are not significantly different, whereas overall morbidity might be similar or possibly improved with MIE. However, the ultimate message is that better data is necessary to claim the benefits of MIE over open esophagectomy. Consequently, Biere et al. published the study protocol on the “TIME” trial or traditional invasive versus minimally invasive esophagectomy, which will be the first prospective, multicenter, randomized study comparing open versus MIE [30]. In this study proposal, patients will be randomized to either traditional open Ivor Lewis esophagectomy or MIE. For the MIE group, THE is the preferred method while the Ivor Lewis esophagectomy (i.e., an intrathoracic anastomosis) will be performed if more of a gastric resection margin is necessary based on location of the tumor.

## 3. Surgical Procedure Considerations

### 3.1. Prone Positioning

Some surgeons have reported on thoracoscopic mobilization of the esophagus in prone position as a potentially more ergonomic approach with less pulmonary morbidity. Palanivelu et al. published a series of 130 patients who underwent MIE with thoracoscopic esophageal mobilization in prone position using a right prone posterior approach, thus allowing for left lung ventilation with possible intermittent ventilation of the right lung. Mean operative time was 220 minutes (range 160 to 450 minutes). Median ICU stay was 1 day (range 1 to 32 days). Postoperative pneumonia rate was only 1.54% [31]. From a respiratory standpoint, the authors advocate prone positioning by arguing for reduction in venous shunt and possibly prevention of postoperative atelectasis, due to allowing of partial intermittent right lung ventilation, as opposed to single (left) lung ventilation. Functional residual capacity and ventilation-perfusion matching are better in prone, compared to supine, position.

Noshiro et al. published their experience with prone positioning during MIE and aggressive mediastinal lymphadenectomy. Compared with left lateral decubitus positioning, prone positioning was associated with lower blood loss and better exposure of the operative field around the left recurrent laryngeal nerve (i.e., identification of the left subclavian artery to allow for dissection around the left recurrent laryngeal nerve). Recurrent laryngeal nerve injury rates were not significantly different between prone and left lateral decubitus-positioned patients. No local recurrences were observed along the left recurrent laryngeal nerve at a median of 22-month followup after surgery [32].

Fabian et al. found in their single institution retrospective comparison of prone versus left lateral decubitus position during thoracoscopic esophageal mobilization that operative times were significantly shorter in the prone group (86 versus 123 min, *P* < 0.01) [33]. Complication rate, number of lymph nodes procured, length of stay, and intraoperative blood loss were not significantly different between prone and left lateral positions.

Jarral et al. addressed the controversy of prone versus left lateral decubitus position during the thoracoscopic phase of three-stage minimally invasive esophagectomy in a review of 31 papers, 7 of which represented the best evidence available. The authors' conclusions were that the studies are small in size, have significant limitations, and do not uniformly demonstrate superiority in outcomes with either technique [34].

### 3.2. Gastric Conduit 

Luketich et al. reported an increase in gastric tip necrosis and anastomotic leaks when a narrower gastric tube (3-4 cm in diameter) was employed; thus, they emphasized the importance of creating a gastric tube of 5-6 cm in diameter [14]. In an editorial on MIE, Wee and Morse also recommend creating a gastric tube of 5 cm in diameter [35]. Additionally, a case series of 77 patients who underwent intended MIE by Berrisford et al. reported a higher than expected gastric tube ischemia rate of 13% using an ideal gastric tube diameter of 4 cm [36].

Based on anecdotal reports of gastric ischemic conditioning and low anastomotic leak rates, Nguyen et al. performed a retrospective comparative study of 81 patients who underwent MIE after laparoscopic gastric ischemic “conditioning” via division of the left gastric artery during staging laparoscopy versus 71 patients who underwent esophagectomy without “conditioning.” There were no significant differences in anastomotic leak rate (11% for conditioning versus 8.5% without conditioning) or stricture rate (30% versus 25%) [37]. In the LOGIC trial, a randomized controlled trial of laparoscopic gastric ischemic conditioning prior to MIE, perfusion of the gastric conduit tip—as measured by laser Doppler fluximetry—was not improved by conditioning, although the number of study patients was only 16 [38].

### 3.3. Esophagogastric Anastomosis

Regarding the technique of esophagogastric anastomosis, Luketich et al. described, as others have, employing a 25 mm EEA stapler via a small gastrotomy [6]. A modification to this was described by Campos et al. in which a circular stapled anastomosis is performed using a transoral anvil. Their study included 37 patients who underwent minimally invasive ILE using this new stapled technique; anastomotic leak rate was 2.7%, and stricture rate was 13.5%, all of which were treated successfully with endoscopic dilations [39]. We previously have described our technique of completely thoracoscopic side-to-side stapled 6 cm anastomosis during minimally invasive ILE by placing the anvil portion of the stapler through the esophagostomy (which is guided by a large bore nasogastric tube or Bougie dilator) and the cartridge through the gastrotomy. In this small study, there were no postoperative strictures [40].

Maas et al. performed a review of 12 studies describing various intrathoracic anastomotic techniques during minimally invasive ILE, including descriptions of handsewn techniques and mostly stapled techniques. The stapled techniques were either transthoracic or transoral, and the transthoracic stapled techniques varied with regard to (1) left lateral decubitus versus prone positioning; (2) circular versus linear stapled; (3) end-to-side versus side-to-side; (4) use of purse string device, handsewn purse string, endostitch, or linear staple gun and Z-stitch for securing the anvil on the proximal esophageal side. Anastomotic leak rates ranged from 0 to 10%, and anastomotic stricture rates ranged from 0 to 27.5% [41].

### 3.4. Management of Leak (OR versus Stent)

Regarding the management of esophagogastric anastomotic leak, minimally invasive options such as endoscopic stenting have been increasingly described. We described our development of an algorithm for the management of endoscopic perforations, due to the need for multiple diagnostic modalities and therapeutic interventions in a short period of time. For esophageal perforations, an initial attempt at esophageal stent placement or clipping was performed if feasible [42, 43]. Nguyen et al. performed a retrospective comparative study of conventional treatments versus endoscopic stenting for esophagogastric anastomotic leaks. Nine patients were treated with conventional therapy, which included neck drainage (*n* = 4), cervical esophagostomy (*n* = 2), thoracoscopic drainage with or without repair or T-tube placement (*n* = 3), and 9 patients were treated with endoscopic stenting with or without percutaneous drainage. Control of leak was seen in 89% versus 100% in the conventional versus endoscopic treatment groups, respectively. The in-hospital or 60-day mortality was 0% for both groups [44]. The above data suggest that nonoperative, endoscopic management of esophageal perforations or esophagogastric anastomotic leaks is safe and feasible and may have comparable outcomes to conventional treatment. A prospective, controlled study is necessary to demonstrate any significant benefit with less invasive, endoscopic interventions.

### 3.5. Quality of Life Parameters

Quality of life is increasingly becoming an important outcome assessment for patients who have undergone esophagectomy. Parameswaran et al. and Nafteux et al. performed a comparative analysis of outcomes, including health-related quality of life (HRQoL) after open versus MIE. At 3 months, postoperative fatigue, general pain, and gastrointestinal pain were all less with MIE [45, 46]. Sundaram et al. evaluated perioperative outcomes, survival, and quality of life (QoL) after open versus MIE at their institution (*n* = 104). Regarding QoL, the authors employed the European Organization for Research and Treatment of Cancer QoL and esophageal module questionnaires, and patients were surveyed more than 1 year after their surgery. There was no significant difference in any of the quality of life parameters between open versus MIE [47]. 

A symptom assessment study was performed by Mehran et al. retrospectively comparing the functional benefits of open esophagectomy with MIE. Propensity matching was used to generate 44 pairs (of open versus MIE). Patients were asked 26 questions about reflux or dumping symptoms using validated questionnaires. The reflux score was slightly worse in the MIE group (*P* = 0.02), and there was no significant difference in the dumping symptoms between open and MIE. Seventy-seven percent in the MIE group compared to 93% in the open group were satisfied with the overall result. Overall, functional outcomes were comparable between open and MIE [48].

### 3.6. Robotic Esophagectomy

Thoracoscopic robot-assisted MIE is increasingly being utilized. Weksler et al. performed a retrospective comparison of 11 patients who underwent thoracoscopic robot-assisted MIE versus 26 who underwent MIE. No significant differences were seen in operative time, blood loss, postoperative complications, duration of mechanical ventilation, or length of stay. No significant advantages were seen with robot-assisted MIE [49]. Galvani et al. at the University of Illinois reported on 18 patients who underwent robotic-assisted THE. Although anastomotic leak occurred in 6 (33%) patients, other parameters such as mean estimated blood loss of 54 mL and low cardiopulmonary morbidity (1 pleural effusion and 2 cases of atrial fibrillation) suggested safety and feasibility of robotic-assisted THE [50]. This study, albeit the largest series published at that time, was limited by its small size and lack of a control group for comparison. Hence, Watson performed a review of robotic-assisted MIE and concluded that the data on robotic-assisted MIE suggest safety, feasibility, and equivalent outcomes compared to open or MIE. However, there is no data to suggest improved outcomes with robotic-assisted MIE in terms of operative morbidity, pain, length of stay, operative time, or total costs [51].

### 3.7. Question of Regionalization, Volume, and Training

Enestvedt et al. reported trends in the management of esophageal cancer, based on provider volume, among 618 esophageal surgeon respondents to a survey. Regarding the adoption and continuation of MIE, a higher proportion of the high volume surgeons—defined as surgeons performing greater than 15 esophagectomies in 1 year—performed MIE compared to the low volume surgeons (26.0 versus 16.5%, *P* = 0.045). The majority of high volume surgeons (87%) practiced in an academic setting and had cardiothoracic training. Cited reasons for discontinuing the practice of MIE were length of case, procedure-related morbidity and mortality, and learning curve [52].

We previously have reported results of a database study on esophagectomies performed in Florida which demonstrated significantly lower risk-adjusted postoperative mortality (OR 0.54, CI 0.32, 0.92) and similar leak rates of 8.2% in centers with higher volume compared with lower volume [53].

## 4. Conclusion

The data suggest that operative mortality is not significantly different between open and MIE, and operative morbidity in a few studies is improved after MIE. Furthermore, comparisons of functional result after open versus MIE without pyloric drainage demonstrate little difference. The data however are severely limited in that the majority of the studies are either retrospective case series, or case-control studies, or meta-analyses of retrospective case-control studies—all limited by both selection and publication bias. Prospective, randomized studies are necessary if any conclusion is to be made about the superiority of one surgical therapy over the other. It is also not clear that robotic-assisted MIE offers any advantages over conventional open or MIE. The data also does not demonstrate superior outcomes as a result of prone positioning or gastric ischemic conditioning. Leaks are increasingly being managed via less invasive, endoscopic stenting with good outcomes, but controlled, prospective studies are necessary to make any conclusions about outcomes after endoscopic stenting versus conventional treatments. The data does certainly support improved outcomes when performed at high volume centers by experienced esophageal surgeons. Overall, MIE is an integral tool that is safe and effective in the surgical management of esophageal cancer, and further study is warranted to determine if it should be the gold standard procedure.

## Figures and Tables

**Table 1:** MIE outcomes in institutional series, case-control studies, and systematic reviews.

Study	*N*	Type	Leak	Pneumonia	RLN injury	Morbidity	Mortality
Institutional series							

Luketich et al. [14]	206	MIE	11.7%	7.7%	3.6%	—	1.4%
Bizekis et al. [15]	50	MIE	6%	—	—	—	6%
Rajan et al. [16]	463	MIE	—	—	—	16%	0.9%
Nguyen et al. [17]	104	MIE	9.6%	—	—	12.5%	2.9%
Ben-David et al. [18]	105	MIE	4%	9%	7%	—	1%
Ben-David et al. [19]	18	MIE	5.6%	16.7%	—	—	5.6%

Systematic reviews or meta-analyses							

Gemmill and McCulloch [20]	1398	MIE	7.7%	13.2%		46.2%	2.3%
Verhage et al. [21]	—	Open		22.9%		60.4%	3.8%
(10 case-control studies)	—	MIE		15.1%		43.8%	1.3%
Nagpal et al. [22]	612	Open	No difference				No difference
(12 case-control studies)	672	MIE	No difference				No difference
Dantoc et al. [23]	—	Open					4.4%
(17 case-control studies)	—	MIE					3%
Sgourakis et al. [24]	1008	Open versus MIE					Total complications lower with MIE
Biere et al. [25]	1061	Open versus MIE					Trends favoring MIE, but not significant

(1 randomized controlled trial and 9 case-control studies)							

Mamidanna et al. [26]	6347	Open				39.2%	4%
	1155	MIE				38%	4.3%

MIE: minimally invasive esophagectomy.

RLN: recurrent laryngeal nerve.
